# Matrix-producing metaplastic breast carcinoma – a rare tumor with heterologous elements

**DOI:** 10.3205/000258

**Published:** 2017-12-06

**Authors:** Sharma Shruti, Fouzia Siraj

**Affiliations:** 1 National Institute of Pathology (ICMR), Safdarjung Hospital Campus, New Delhi, India

**Keywords:** chondromyxoid areas, matrix-producing carcinom, metaplastic breast carcinoma

## Abstract

Metaplastic breast carcinomas are ductal carcinomas that undergo metaplasia into non-glandular growth patterns. They are very rare and account for less than 1% of all invasive breast carcinomas. Matrix-producing carcinoma is an extremely rare and aggressive subtype of metaplastic breast carcinoma that is characterized by a ductal carcinomatous component with direct transition to areas with cartilaginous/osseous differentiation without an intervening spindle cell element. It has a better prognosis than metaplastic carcinoma. Even though these tumors are composed of a mixture of infiltrating ductal carcinomas and areas of heterologous stroma, each of which behaves aggressively individually, these composite tumors have a better 5-year survival rate with rare nodal metastasis.

Immunohistochemically, they are positive for keratin, epithelial membrane antigen and S100. The tumor, which is matrix-producing, is S100-reactive and nonreactive for cytokeratin. They are usually hormone receptor-negative. The average age of these patients is approximately 58 years. Since these tumors are usually triple-negative, chemotherapy after surgery is the mainstay of therapy, using either mastectomy or local excision.

Our report highlights this rare entity in a 55-year-old female patient with matrix-producing metaplastic breast carcinoma. Its distinctive histological features and peculiar clinical behavior warrants clear knowledge about this unique entity.

## Zusammenfassung

Metaplastische Mammakarzinome sind duktale Karzinome, die Metaplasie zu nicht-glandulären Wachstumsmustern durchlaufen. Sie sind sehr selten und machen weniger als 1% aller invasiven Mammakarzinome aus. Das Matrix-produzierende Karzinom ist ein extrem seltener und aggressiver Subtyp eines metaplastischen Mammakarzinoms, das durch eine duktale karzinomatöse Komponente mit direktem Übergang in Bereiche mit knorpeliger/knöcherner Differenzierung ohne dazwischenliegendes Spindelzellelement gekennzeichnet ist. Es hat eine bessere Prognose als das metaplastische Karzinom. Obwohl diese Tumore aus einer Mischung von infiltrierenden duktalen Karzinomen und Bereichen von heterologem Stroma bestehen, von denen jedes sich einzeln aggressiv verhält, haben diese zusammengesetzten Tumore eine bessere 5-Jahres-Überlebensrate mit seltener Lymphknotenmetastase.

Immunhistochemisch sind sie positiv für Keratin, epitheliales Membranantigen und S-100. Matrix-produzierende Tumore sind S-100-reaktiv und nicht reaktiv für Cytokeratin. Sie sind normalerweise Hormonrezeptor-negativ. Das Durchschnittsalter der Patientinnen beträgt ca. 58 Jahre. Da diese Tumoren in der Regel dreifach negativ sind, ist die Chemotherapie nach der Operation das Hauptelement der Therapie, entweder mit Mastektomie oder lokaler Exzision.

Unser Bericht beschreibt den Fall einer 55-jährigen Patientin mit Matrix-produzierendem metaplastischen Brustkarzinom, die charakteristischen histologischen Merkmalen und das besondere klinische Verhalten dieses seltenen Krankheitsbildes.

## Introduction

 Metaplastic breast carcinomas (MBC) are very rare infiltrating ductal carcinomas (IDC) that undergo metaplasia into non-glandular growth patterns. They account for <1% of all invasive BC, have a poor prognosis and high incidence of recurrence [1]. Matrix-producing carcinomas (MPC) are a very rare subtype of MBC with a better prognosis and comprise <0.1% of invasive BC. They are characterized by nonaggressive behavior and direct transition of carcinomatous component to cartilaginous/osseous matrix without an interspersed spindle cell component [2]. This case is being reported not only for its rarity but also highlights its better prognosis and hence, the need for its recognition.

## Case presentation

**Figure 1:**
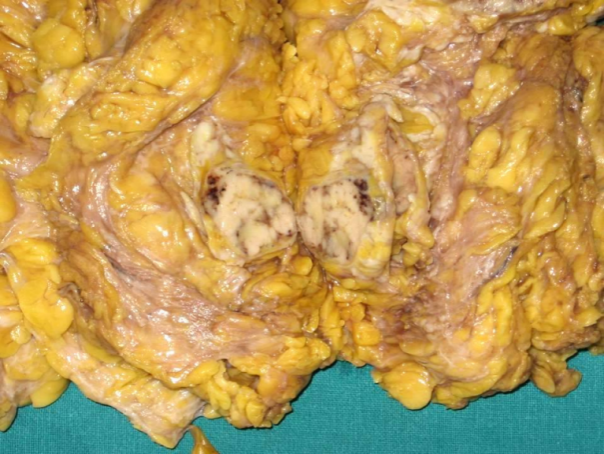
Gross photograph of mastectomy specimen with cut surface revealing a fibrous, grey-white, partially encapsulated tumor

**Figure 2:**
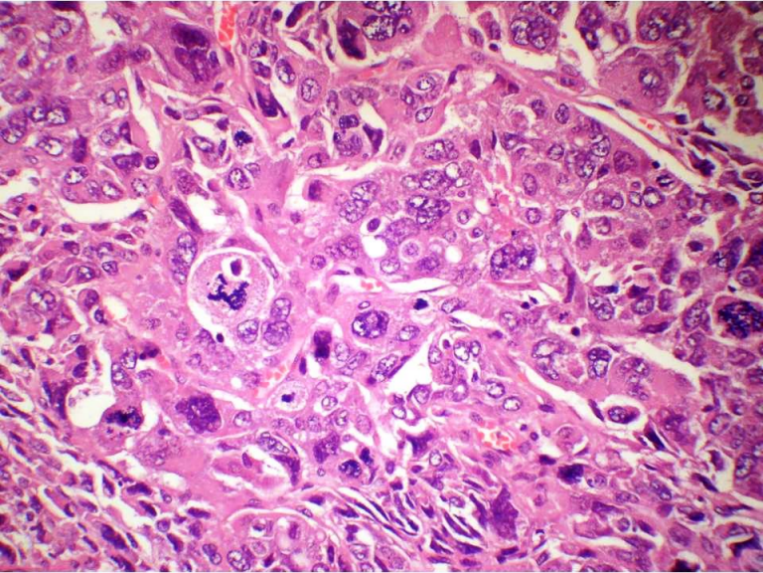
Photomicrograph showing IDC with marked nuclear pleomorphism and atypical mitotic figures (H&E 400X)

**Figure 3:**
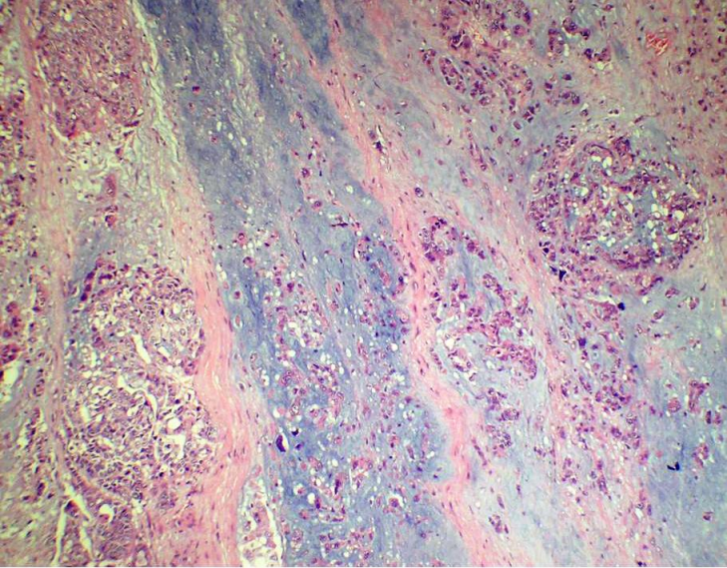
Photomicrograph showing tumor cells scattered singly, in groups and cords within a chondromyxoid matrix with atypical nuclei and eosinophilic cytoplasm (H&E 200X)

**Figure 4:**
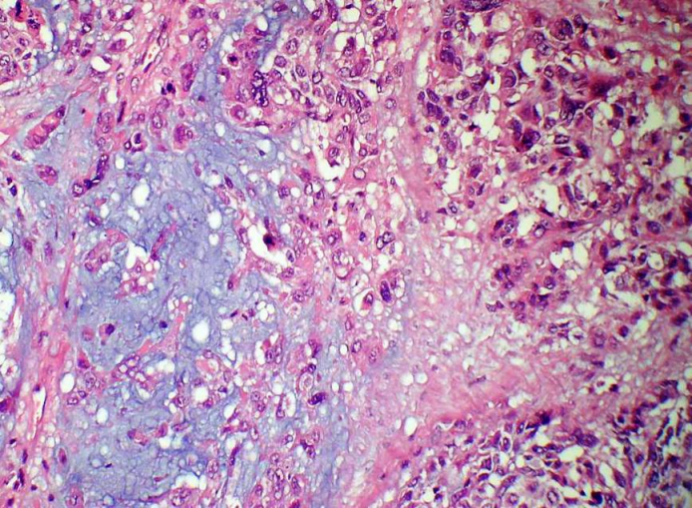
Photomicrograph showing invasive ductal carcinoma with an abrupt transition to chondromyxoid matrix without an intervening spindle cell component (H&E 200X)

**Figure 5:**
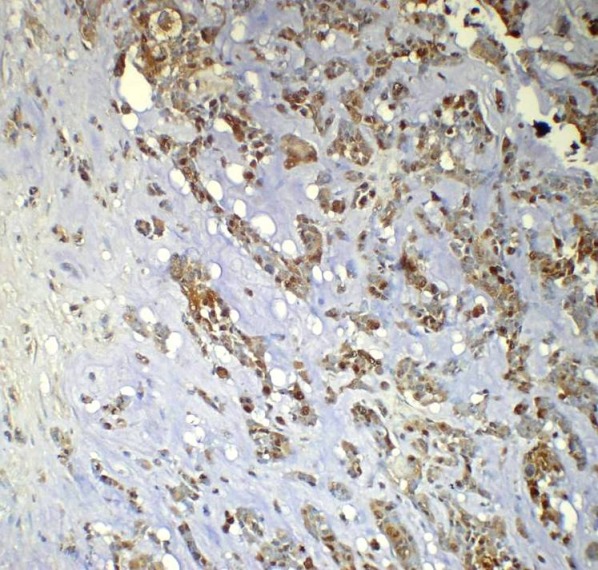
Immunohistochemical profiling: Matrix-producing tumor cells showing positive immunoexpression for S100 and negative for cytokeratin (IHC 200X)

**Figure 6:**
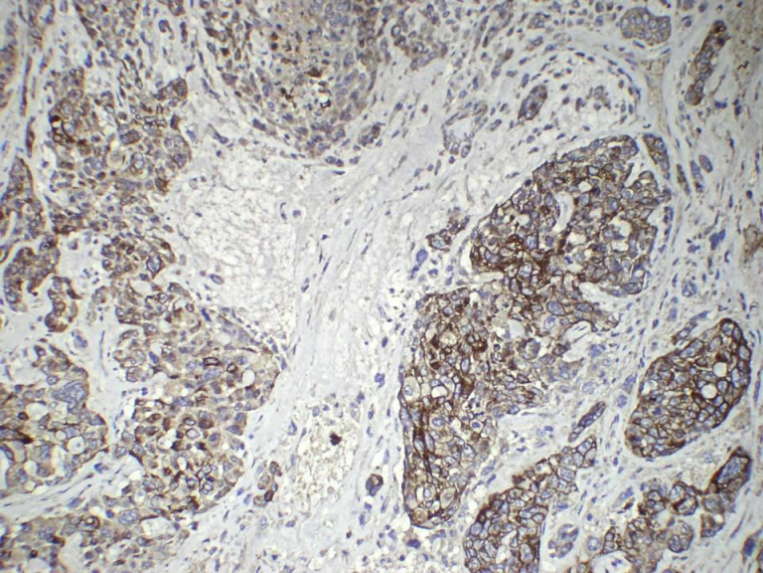
Immunohistochemical profiling: Infiltrating ductal carcinoma cells showing positive immunoexpression for cytokeratin and negative for S100 (IHC 200X)

A 55-year-old female presented with a mass in the medial aspect of the right breast. Clinically, a single 4×3 cm, non tender, firm, and mobile lump was palpable in the right breast in the lower inner quadrant without nipple retraction or skin adhesion. This was confirmed mammographically. The left breast and axillary lymph nodes were uninvolved. Hematological, biochemical, and metastatic workup were normal. Incisional biopsy of the lump was diagnosed as IDC with chondroid areas and the patient was staged as T2N0M0 on TNM classification. Subsequently, modified radical mastectomy with axillary node dissection was performed. Grossly, cut surface revealed a fibrous, grey-white, partly encapsulated 3.5×3 cm tumor with mucoid and glistening areas (Figure 1 (Fig. 1)). Microscopically, the tumor had two distinct patterns; high grade IDC (Figure 2 (Fig. 2)) and tumor cells scattered singly, in groups and cords within a chondromyxoid matrix. These were medium- to large-sized, chondrocyte-like oval-shaped with atypical nuclei and eosinophilic cytoplasm. Mucoid to overtly cartilaginous areas were identified in the matrix (Figure 3 (Fig. 3)). The chondromyxoid matrix was multifocal, of high grade with abrupt transition between IDC component and metaplastic areas (Figure 4 (Fig. 4)). No lymphovascular invasion was identified, surgical margins and lymph nodes were uninvolved by carcinoma. Due to the presence of IDC component and chondroid metaplastic areas, the diagnosis of matrix-producing metaplastic carcinoma of breast was rendered. This was further confirmed by immunohistochemical profiling. The tumor was negative for hormone receptors and HER2/neu. IDC cells stained positive for cytokeratin and negative for S100 (Figure 5 (Fig. 5)). MPC areas revealed immunoexpression for S100 and were negative for cytokeratin (Figure 6 (Fig. 6)). The patient was given six cycles of chemotherapy and local radiation therapy and is well two years post surgery.

## Discussion

MPC, a rare subtype of MBC, presents at an average age of 58 years as a rapidly enlarging, painless, well-delineated mass with a better prognosis [3]. The cellular origin of the MPC remains unclear with ultrastructural analysis supporting the evidence that tumor cells are of both epithelial and myoepithelial derivation. Myoepithelial cells differentiate along mesenchymal lines and produce a gamut of matricial appearances [2]. Few studies have suggested a neoplastic transformation of multipotent stem cells. The atypical metaplastic cartilaginous matrix also called “high-grade matrix” is known to have an adverse prognosis. But Downs-Kelly et al. found no correlation between matrix grade and tumor recurrence [4]. Though MPCs usually present as solitary masses, Wargotz and Norris reported multiple discrete tumor nodules on gross assessment following excision. They also documented the association of atypical cartilaginous metaplasia with aggressive tumor progression [5]. MBCs have similar to or more aggressive behavior than IDC matched for patient age, stage and tumor grade [4]. They present at an advanced stage, recur locally and are aggressive with a poor outcome. Important factors in determining outcome are tumor size and mesenchymal component with worse survival rate seen in patients with tumor >5 cm. But high proportion of matrix as seen in MPC and spindle cell MBC has a more favorable outcome [6]. 

Clinically and mammographically they resemble IDC which are firm-to-hard, nodular, and circumscribed. In majority, diagnosis is difficult on cytology/biopsy due to varied tumor presentation [1]. Histological examination of the excised tissue helps in distinguishing true chondrosarcoma of the breast and MPC to yield a differential diagnosis. Immunohistochemically ductal component is positive for cytokeratin while matrix producing component is S100-reactive and nonreactive for cytokeratin [7]. Since about 28% of MBC harbor epidermal growth factor receptor (EGFR) amplification and lack hormone receptors, some patients might benefit from novel therapies like EGFR tyrosine kinase inhibitors targeting EGFR [3]. Since majority of these tumors are hormone receptor- and HER2/neu-negative, chemotherapy after surgery is the mainstay of treatment. In our case, the tumor cells in both the metaplastic and ductal areas were triple-negative in accordance with the study by Gibson et al. [8].

MPC rarely metastasize via the lymphatic system and prefer the hematogenous route [1]. Lymph nodes in our patient were disease-free. Axillary lymph node metastasis varies from 6% to 25%. The 5-year survival rate is 70% following mastectomy and 50% after local excision [9]. Large poorly differentiated tumors, nodal metastasis and atypical cartilaginous metaplasia with diffuse cellularity indicate poor prognosis [9]. The prognosis of MBC is considered worse than same stage breast carcinoma of no special type [5]. 

 The role of radiotherapy and chemotherapy is not clear; hence, surgery is the treatment of choice [1]. Due to intratumoral heterogeneity, MBC are chemoresistant. For hormone receptor-negative tumors, hormone therapy is unnecessary. Literature search reveals very few and inconsistent data with no standard treatment for this subgroup of breast cancer. Recently few clinical trials have documented the role of targeted gene therapy following genetic profiling [1].

In our case, even in the absence of axillary lymph node metastasis, aggressive chemotherapy was given due to the high-grade nature of IDC component and high-grade matrix leading to a higher probability of distant metastases. 

Even though these tumors are composed of a mixture of high-grade IDC and heterologous stroma, each of which behaves aggressively, these composite tumors have a better 5-year survival rate with rare nodal metastasis. As regards this peculiarity, identifying MPC is crucial as its prognosis is superior to other MBCs and hence, it deserves a separate position in tumor classification [9]. In future it would be necessary to study a larger number of patients with MPC and further elucidate the clinicopathological characteristics of this malignancy.

## Notes

### Competing interests

The authors declare that they have no competing interests.

## References

[1] Rossi L, Paglicci C, Caprio G, Barberi S, Ranieri E, Zancla S, Rengo M, Pagni P (2013). Matrix-producing carcinoma of the breast: a case report. Case Rep Oncol.

[2] Sari A, Çakalagaoğlu F, Altinboğa AA, Kucukzeybek BB, Calli A, Atahan MK (2015). Cytopathological features of matrix-producing carcinoma of the breast. J Cytol.

[3] Bhosale SJ, Kshirsagar AY, Sulhyan SR, Sulhyan SR, Jagtap SV (2013). Matrix-producing metaplastic breast carcinoma - a rare malignancy. Am J Case Rep.

[4] Downs-Kelly E, Nayeemuddin KM, Albarracin C, Wu Y, Hunt KK, Gilcrease MZ (2009). Matrix-producing carcinoma of the breast: an aggressive subtype of metaplastic carcinoma. Am J Surg Pathol.

[5] Ayar S, Dyess DL, Carter E (2010). Matrix-producing carcinoma: a rare variant of metaplastic breast carcinoma with heterologous elements. Breast J.

[6] Liu LY, Sheng SH, Zhang ZY, Xu JH (2015). A case of matrix-producing carcinoma of the breast with micoglandular adenosis and review of literature. Int J Clin Exp Pathol.

[7] Kusafuka K, Muramatsu K, Kasami M, Kuriki K, Hirobe K, Hayashi I, Watanabe H, Hiraki Y, Shukunami C, Mochizuki T, Kameya T (2008). Cartilaginous features in matrix-producing carcinoma of the breast: four cases report with histochemical and immunohistochemical analysis of matrix molecules. Mod Pathol.

[8] Gibson GR, Qian D, Ku JK, Lai LL (2005). Metaplastic breast cancer: clinical features and outcomes. Am Surg.

[9] Wargotz ES, Norris HJ (1989). Metaplastic carcinomas of the breast. I. Matrix-producing carcinoma. Hum Pathol.

[10] Mardi K, Sharma J (2008). Matrix-producing mammary carcinoma: a rare breast tumor. Indian J Pathol Microbiol.

